# Dietary inclusion of a partially defatted black soldier fly (*Hermetia illucens*) larva meal in low fishmeal-based diets for rainbow trout (*Oncorhynchus mykiss*)

**DOI:** 10.1186/s40104-021-00575-1

**Published:** 2021-04-16

**Authors:** Christian Caimi, Ilaria Biasato, Giulia Chemello, Sara Bellezza Oddon, Carola Lussiana, Vanda Maria Malfatto, Maria Teresa Capucchio, Elena Colombino, Achille Schiavone, Francesco Gai, Angela Trocino, Alberto Brugiapaglia, Manuela Renna, Laura Gasco

**Affiliations:** 1 Department of Agricultural, Forest and Food Sciences, University of Turin, Largo P. Braccini 2, 10095 Grugliasco, TO Italy; 2 Department of Veterinary Sciences, University of Turin, Largo P. Braccini 2, 10095 Grugliasco, TO Italy; 3 Institute of Science of Food Production, National Research Council, Largo P. Braccini 2, 10095 Grugliasco, TO Italy; 4 Department of Comparative Biomedicine and Food Science, University of Padua, Viale dell’Università 16, 35020 Legnaro, PD Italy

**Keywords:** Apparent digestibility coefficient, Chemical and physical characteristics of fillets, Fatty acid profile, Fishmeal substitution, *Hermetia illucens* meal, Histopathology, Performance

## Abstract

**Background:**

Recent investigations highlighted that *Hermetia illucens* (BSF) larva meal can be a valuable alternative protein source for aquafeed production. In this study, in substitution of fishmeal, we used increasing inclusion levels of a partially defatted BSF larva meal in low fishmeal-based diets for rainbow trout (*Oncorhynchus mykiss* Walbaum) and we evaluated the related implications in terms of growth performance, physical characteristics, proximate and fatty acid (FA) compositions of the fillets, gut and liver histology, and diet digestibility. In a 131-day trial, 576 fish (100.1 ± 9.29 g) were randomly allotted to 24 tanks (24 fish/tank, 4 replicates/treatment). Six experimental diets were produced to have partial replacement of fishmeal (0, 10%, 20%, 30%, 40% and 50%) by increasing levels of BSF meal (0% [BSF0], 3% [BSF3], 6% [BSF6], 9% [BSF9], 12% [BSF12] and 15% [BSF15] on as fed basis, respectively).

**Results:**

No differences were observed among the treatments for all the considered growth performance parameters. The viscero and hepato-somatic indexes showed significant differences among the treatments, with the highest values observed in the BSF15 group. No differences were recorded in terms of fillet’s physical characteristics, dry matter (DM), crude protein (CP) and ether extract (EE) contents. Total saturated and monounsaturated FA increased, while polyunsaturated FA (particularly n-3 FA) decreased while increasing the HI meal inclusion in the diet. Histopathology of liver and gut was not affected, whereas, in posterior gut, villi were higher in BSF6 and BSF9 compared to BSF3 fish. The apparent digestibility of DM, CP, EE and gross energy did not vary among the treatments.

**Conclusions:**

These results suggest that a partially defatted BSF meal could be included up to 15% in low fishmeal-based diets for rainbow trout with no adverse effects on growth performance, fillet’s physical characteristics, gut and liver health, and diet digestibility. On the contrary, the fillet FA composition worsened while increasing the level of BSF meal in the diet.

## Background

In the next few years, there will be an increase in the global demand of protein because of the constant increase in the world population [1]. Aquaculture has the fastest growing in the food production, with an average annual rate above 5.5% per year [2] and, for this reason, is considered as one of the livestock sectors able to support the global demand of animal products [3]. The growing in fish production leads to an increase in the demand of aquaculture feeds. For many years, fishmeal (FM) has been the preferred protein source for the production of aquafeeds due to its valuable content of protein and fatty acids (FA), amino acid profile, high digestibility and palatability [4]. However, the constant increase of the demand of aquaculture feeds has led to a rapid growth in the FM price and to a negative impact on the marine ecosystem [5]. In order to maintain the correct percentage of protein to meet the nutritional requirements of fish, in the last 20 years FM has been partially replaced with alternative raw materials, such as vegetable protein sources and processed animal proteins (PAPs), leading to a reduction in the dietary FM inclusion in the feeds.

Recently, in order to replace FM, the attention has been focused on the use of insect-derived PAPs. Insects can be used to produce high quality ingredients, rich in protein and fat, starting from waste biomass and with low environmental impact [6, 7]. One of the insect species with the highest potential to be used in fish feeds is the black soldier fly (*Hermetia illucens* L.) (BSF). The larvae of this fly can be reared on low value organic waste, with low water demand and generating low greenhouse gas emissions [8]. Generally, the meals obtained from BSF show a protein content ranging from 37% to 63%, and a fat content from 7% to 39% on a dry matter (DM) basis [9, 10].

Despite the nutritional value of *BSF meal,* its successful inclusion level in aquafeeds depends also on their effect on gut health and liver integrity, which are fundamental for nutrient digestion and absorption and thus for growth performances [11]. For this reason, gut histomorphometry and liver histopathology are usually taken into account when alternative ingredients are investigated in animal nutrition [12]. Nutritional studies on the total and partial substitution of FM with BSF meal have been previously conducted in rainbow trout (*Oncorhynchus mykiss* Walbaum). A recent study by Cardinaletti et al. [12] showed that in a control diet containing 42% of FM, the 50% of the FM could be replaced including a dietary inclusion up to 21% of a full-fat BSF meal without negative effects on the growth performance of rainbow trout. However, a decrease in villus height and an increase in liver lipid accumulation was observed in trout fed BSF meal. Compared to a control diet with a FM dietary inclusion of 60%, Renna et al. [13] showed that an inclusion up to 40% of partially defatted BSF meal could replace up to 50% of FM without any adverse effects on growth performance, gut and liver histomorphometry. On the contrary, studies by Dumas et al. [14] and St-Hilaire et al. [15], using a defatted BSF meal (up to 26.4% of inclusion) and a full-fat BSF meal (up to 29.8% of inclusion), respectively, showed a worsening of the growth performance of trout at increasing BSF inclusion levels. The growth performance and the gut and liver histopathology reported in these studies showed contradictory results probably due to several factors, such as the nutritional composition and inclusion level of the insect meal, and fish size.

As we can see in literature, most of the studies performed in fish nutrition replacing FM with BSF meal have been conducted using control diets with high levels of FM, usually higher than 30% [13, 16–20]. However, due to the current price of FM, only 10–20% of FM is currently included in commercial diets [2, 21].

Therefore, the aim of this study was to determine the potential of six inclusion levels of a partially defatted BSF larva meal as a partial replacer of FM in low FM-based diet, on growth performance, somatic indexes, fillet’s physical characteristics, proximate composition and FA profile, histopathological investigation of gut and liver, and digestibility in rainbow trout.

## Methods

The experimental protocol was designed according to the guidelines of the current European Directive on the protection of animals used for scientific purposes (2010/63/EU) and approved by the Ethical Committee of the University of Turin (Italy) (protocol n° 143811). The trial was carried out at the Experimental Facility of the Department of Agricultural, Forest and Food Sciences (DISAFA) of the University of Turin (Italy).

### Experimental diets

Six experimental diets were formulated to be isonitrogenous (crude protein – CP: about 45.8 g/100 g as fed), isolipidic (ether extract – EE: about 15.2 g/100 g as fed), and isoenergetic (gross energy – GE: about 22.6 MJ/kg as fed). The six diets were obtained including, as fed basis, increasing levels of a partially defatted BSF larva meal – 0, 3%, 6%, 9%, 12% and 15% – corresponding to a substitution of 0% (BSF0), 10% (BSF3), 20% (BSF6), 30% (BSF9), 40% (BSF12) and 50% (BSF15) of FM. The BSF larva meal used in this study was provided by MUTATEC (Caumont-sur-Durance, France). The larvae had been raised on plant by-products and partially defatted using a mechanical process. Unfortunately, no other information was given by the producer about either the rearing substrate or the processing methodologies, as this information is considered confidential. The experimental diets were prepared at the Experimental Facility of DISAFA. All the ground ingredients and oils were individually weighed (KERN PLE-N v.2.2; KERN & Sohn GmbH, Balingen-Frommern, Germany; d: 0.01) and mixed with a blender (Brevetti S.A.G.A., Milano, Italy). To facilitate the pelleting process, an amount of 250 to 500 mL/kg of water was added to the mixture. The pelletizing was performed using a meat grinder (LABOR 32; Rheninghaus Factory, San Mauro Torinese, Italy). The pellets (3.0 mm) were subsequently dried (50 °C for 48 h) and stored in black bags at − 20 °C until used. The ingredients of the experimental diets are reported in Table 1.

**Table 1:** Ingredients and proximate composition of the experimental diets and BSF larva meal

	BSF	BSF0	BSF3	BSF6	BSF9	BSF12	BSF15
Ingredients, %							
Fishmeal^a^		20.0	18.0	16.0	14.0	12.0	10.0
*Hermetia illucens* larva meal^b^		–	3.0	6.0	9.0	12.0	15.0
Wheat gluten		13.0	13.0	13.0	13.0	13.0	13.0
Soybean meal		20.0	20.0	20.0	20.0	20.0	20.0
Swine haemoglobin		9.2	9.0	8.8	8.6	8.4	8.2
Wheat starch		23.4	22.6	21.8	21.0	20.2	19.4
Fish oil		7.0	7.0	7.0	7.0	7.0	7.0
Soybean oil		7.0	7.0	7.0	7.0	7.0	7.0
Minerals^c^		0.25	0.25	0.25	0.25	0.25	0.25
Vitamins^d^		0.20	0.20	0.20	0.20	0.20	0.20
Chemical composition ^e^							
Dry matter, g/100 g	94.0	97.2	97.2	96.9	96.8	96.9	96.6
Ash, g/100 g as fed	10.2	5.8	5.9	5.8	5.7	5.5	5.5
Crude protein, g/100 g as fed	56.9	45.6	46.1	45.6	46.0	45.7	46.1
Ether extract, g/100 g as fed	7.0	14.9	15.9	15.8	15.7	14.6	14.3
Gross energy, MJ/kg as fed	20.2	22.4	22.7	22.6	22.7	22.7	22.6
Chitin, g/100 g	6.3	–	0.18	0.37	0.56	0.75	0.93

*Abbreviations: BSF Hermetia illucens*. ^a^ Purchased from Skretting Italia S.p.A. (Località Vignetto, 17-37060 Mozzecane VR, Italy). Proximate composition (g/100 g, as fed): DM 91.0, CP 67.6, EE 8.3, Ash 16.4. ^b^ Provided by MUTATEC (Caumont-sur-Durance, France). ^c^ Mineral mixture (g/kg or mg/kg diet): dicalcium phosphate, 500 g; calcium carbonate, 215 g; sodium salt, 40 g; potassium chloride, 90 g; magnesium chloride, 124 g; magnesium carbonate, 124 g; iron sulphate, 20 g; zinc sulphate, 4 g; copper sulphate, 3 g; potassium iodide, 4 mg; cobalt sulphate, 20 mg; manganese sulphate, 3 g; sodium fluoride, 1 g (purchased from Granda Zootecnici S.r.l., Cuneo, Italy). ^d^ Vitamin mixture (IU/kg or mg/kg diet): *DL*-α-tocopherol acetate, 60 IU; sodium menadione bisulphate, 5 mg; retinyl acetate, 15,000 IU; *DL*-cholecalciferol, 3000 IU; Stay C Roche (vitamin C), 90 mg; thiamin, 15 mg; riboflavin, 30 mg; pyridoxine, 15 mg; vitamin B_12_, 0.05 mg; nicotinic acid, 175 mg; folic acid, 500 mg; inositol, 1000 mg; biotin, 2.5 mg; calcium panthotenate, 50 mg (purchased from Granda Zootecnici S.r.l., Cuneo, Italy). ^e^ Values are reported as mean of duplicate analyses

### Chemical analyses of BSF meal and experimental diets

The proximate composition and the energy level of the BSF meal and experimental diets were measured in duplicate at the DISAFA laboratories. Feed samples were ground using a cutting mill (MLI 204; Bühler AG, Uzwil, Switzerland) and analysed for DM (AOAC #934.01), CP (AOAC #984.13) and ash (AOAC #942.05) contents according to AOAC International [22]; EE (AOAC #2003.05) was analysed according to AOAC International [23]. The GE content was determined using an adiabatic calorimetric bomb (C7000; IKA, Staufen, Germany). The chitin content of BSF meal was estimated according to Finke [24] by correction considering the AA content of the acid detergent fiber (ADF) fraction and assuming the remainder of the ADF fraction is chitin. Due to the presence of vegetable raw material in the diets, the method proposed by Finke [24] cannot be applied to calculate the chitin content of the diets. For this reason, the amount of chitin of the experimental diets was estimated based on the chitin content of the BSF meal and its inclusion level in the diets.

The AA determination of BSF larva meal and experimental diets was performed according to the method described in De Marco et al. [25]. After a 22-h hydrolysis step in 6 mol/L HCl at 112 °C under a nitrogen atmosphere, the AA content in hydrolysate was determined by means of HPLC after postcolumn derivatization. Performic acid oxidation occurred prior to acid hydrolysis for methionine and cysteine. Tryptophan was not determined. The AA composition is shown in Table 2.

**Table 2:** Amino acid (AA) concentration (g/100 g of protein) of BSF meal and experimental diets

	BSF	BSF0	BSF3	BSF6	BSF9	BSF12	BSF15
Essential AA							
Arginine	3.9	5.3	5.2	5.2	5.1	5.0	5.0
Histidine	2.2	2.8	2.8	2.8	2.8	2.7	2.9
Isoleucine	3.3	3.5	3.4	3.4	3.3	3.3	3.3
Leucine	5.2	7.7	7.5	7.5	7.3	7.2	7.1
Lysine	3.8	5.9	5.6	5.5	5.3	5.2	5.0
Methionine	2.6	2.5	2.5	2.5	2.5	2.5	2.6
Cysteine	1.3	1.3	1.2	1.1	1.1	1.0	1.3
Phenylalanine	3.0	4.6	4.6	4.5	4.5	4.4	4.3
Tyrosine	4.8	3.0	3.0	3.1	3.1	3.2	3.2
Threonine	3.1	7.6	7.6	7.6	7.6	7.6	7.6
Valine	4.9	3.5	3.5	3.4	3.4	3.3	3.3
Non-essential AA							
Alanine	6.2	4.8	4.8	4.8	4.8	4.8	4.8
Aspartic acid	6.7	8.1	7.9	7.9	7.7	7.7	7.6
Glycine	4.2	2.7	2.8	2.9	3.0	3.1	3.2
Glutamic acid	8.8	16.0	15.9	16.1	16.1	16.3	16.2
Proline	5.5	8.5	8.2	8.1	7.8	7.6	7.4
Serine	3.7	3.7	3.6	3.7	3.6	3.6	3.6

*Abbreviations*: *BSF Hermetia illucens*

A combined direct trans-esterification and solid-phase extraction was carried out for the determination of the fatty acid profile of the BSF meal and experimental diets, using eptadecanoic acid as internal standard, as reported in Dabbou et al. [26]. Fatty acid methyl esters (FAME) were separated, identified and quantified as reported in Dabbou et al. [27]. The results are expressed as mg/100 g DM and are reported in Table 3.

**Table 3:** Fatty acid profile (mg/100 g DM) of BSF larva meal and experimental diets

	BSF	BSF0	BSF3	BSF6	BSF9	BSF12	BSF15
C10:0	67.8	43.8	60.3	73.4	91.0	49.7	63.2
C12:0	1898.7	6.94	64.7	101.1	177.9	228.4	291.4
C14:0	483.0	413.6	436.2	446.7	465.6	450.9	475.8
C15 *iso*	4.51	13.2	14.3	13.5	13.3	13.4	13.4
C15 *aiso*	5.74	4.62	4.63	4.54	4.12	4.24	4.34
C14:1 *c-*9 + C15:0	27.0	39.0	41.3	41.2	40.8	39.0	41.1
C16 *iso*	1.90	1.50	3.19	1.92	2.34	2.16	2.10
C16:0	1111.4	1901.7	1980.2	2072.9	2058.6	1966.5	2060.0
C17 *iso*	2.83	37.1	40.8	31.5	29.4	29.1	30.0
C17 *aiso*	29.8	11.5	18.6	7.66	4.75	5.85	6.20
C16:1 *c-*9	174.5	488.8	504.4	365.8	281.3	287.0	316.5
C17:1 *c-*9	8.16	14.9	18.5	10.3	8.06	8.38	9.06
C18:0	246.8	548.9	562.0	553.9	537.9	513.9	538.7
C18:1 *t*	14.5	11.0	11.0	9.43	9.24	9.13	10.9
C18:1 *c-*9	1216.0	2078.2	2171.7	1658.9	1278.1	1332.4	1530.6
C18:1 *c-*11	27.8	273.8	281.1	209.8	161.8	162.4	179.4
C18:1 *c-*12	0.32	6.22	3.49	1.25	1.43	1.00	1.16
C18:1 *c-*14 + *t-*16	4.42	11.41	8.85	5.38	4.79	4.16	5.01
C18:2 n-6	403.4	2381.2	2486.6	533.1	398.4	387.7	418.5
C18:3 n-3	26.0	241.3	244.8	30.4	24.3	22.7	21.7
C18:3 n-6	1.56	10.0	10.1	1.89	2.05	1.01	0.28
C20:0	20.0	34.6	34.2	40.0	35.1	37.7	41.8
C20:1 *c-*9	4.34	67.1	65.4	55.3	44.5	43.7	47.1
C20:1 *c-*11	n.d.	2.02	2.08	0.10	0.54	0.19	0.35
C20:2 n-6	n.d.	62.3	59.3	6.37	4.53	4.75	3.91
C20:3 n-6	n.d.	2.44	3.49	0.60	0.17	0.87	0.33
C20:3 n-3	n.d.	13.4	8.40	4.90	2.89	3.22	2.89
C20:4 n-6	1.11	25.3	24.0	4.28	2.75	2.76	1.89
C20:5 n-3	n.d.	298.8	275.8	47.3	35.6	35.9	30.00
C22:0	11.5	31.9	26.7	27.8	28.4	26.6	26.4
C22:1 n-9	n.d.	8.39	8.30	5.87	4.31	4.63	4.38
C22:5 n-3	n.d.	32.9	29.7	2.15	1.47	1.70	2.88
C22:6 n-3	n.d.	119.3	151.5	35.9	23.7	21.9	19.8
Σ SFA	3866.0	3020.3	3205.5	3357.2	3435.2	3312.8	3538.4
Σ MUFA	1450.0	2961.7	3075.0	2322.1	1794.1	1853.0	2104.5
Σ PUFA	436.6	3186.9	3293.7	666.8	495.8	482.4	502.2
Σ PUFA / Σ SFA	0.11	1.06	1.03	0.20	0.14	0.15	0.14
Σ n-3	26.0	705.8	710.2	120.5	87.9	85.3	77.3
Σ n-6	406.0	2481.2	2583.5	546.3	407.9	397.1	424.9
Σ n-3 / Σ n-6	0.06	0.28	0.27	0.22	0.22	0.21	0.18
TFA	5797.4	9236.9	9655.7	6405.3	5779.0	5702.8	6201.1

*Abbreviations*: *DM* dry matter, *BSF Hermetia illucens*, *c cis*, *t trans*, *SFA* saturated fatty acids, *MUFA* monounsaturated fatty acids, *PUFA* polyunsaturated fatty acids, *TFA* total fatty acids, *n.d.* not detected. Values are reported as mean of duplicate analyses

All the chemical analyses of feed were performed in duplicate.

### Fish and rearing conditions

A 131-day growth trial was carried out with rainbow trout purchased from a private fish hatchery (“Troticoltura Bassignana”, Cuneo, Italy). An acclimatization period of two weeks was provided during which the fish were fed a commercial diet (42 g/100 g as fed of CP; 22 g/100 g as fed of EE; Skretting Italia Spa, Mozzecane (VR), Italy). Then a total of 576 fish were lightly anesthetised (60 mg/L MS-222 - PHARMAQ Ltd., Fordingbridge, Hampshire, UK), individually weighed (mean individual initial body weight – iIBW: 100.1 ± 9.29 g; KERN PLE-N v.2.2; KERN & Sohn GmbH, Balingen-Frommern, Germany; d: 0.01) and randomly divided into 24 fiberglass 200-L tanks (four replicate tanks per diet, twenty-four fish per tank). Artesian well water (constant temperature of 13 ± 1 °C) was supplied in flow-through open system with each tank having a water inflow of 8 L/min. Dissolved oxygen was measured every week and ranged between 7.6 and 8.7 mg/L. Feed was distributed by hand twice a day, six days per week (number of feeding days = 121). The fish were fed 1.4% of the tank biomass. Feed intake was checked at each administration and feed administration was stopped as soon as the fish stopped eating. In order to update the daily quantity of feed, the fish were weighed in bulk every 14 days. Mortality was checked every day.

### Growth performance

At the end of the trial, after 24 h of fasting, all the fish were lightly anesthetised and individually weighed. The following performance indexes were calculated:

- Mortality (%) = (number of dead fish / initial number of fish) × 100
- Individual weight gain (iWG, g) = iFBW (average individual final body weight, g) – iIBW (average individual initial body weight, g)
- Specific growth rate (SGR, %/d) = [(InFBW − InIBW) / number of feeding days] × 100
- Feed conversion ratio (FCR) = total feed supplied (g, DM) / WG (g)
- Protein efficiency ratio (PER) = WG (g) / total protein fed (g DM).

Individual initial and final body weight were used to calculate the iWG while SGR, FCR and PER were calculated per tank.

### Somatic indexes, carcass yield and coefficient of fatness

At the end of the trial, twenty-eight fish per treatment (seven fish per tank) were sacrificed by over anaesthesia (MS-222, PHARMAQ Ltd., Fordingbridge, Hampshire, UK; 500 mg/L). The fish were individually weighed and then slaughtered to calculate the carcass yield (CY), the hepatosomatic index (HSI), the viscerosomatic index (VSI), and the coefficient of fatness (CF). The following formulas were used:

- CY (%) = [total weight without gut and gonad (g) / fish weight (g)] × 100
- HSI (%) = [liver weight (g) / fish weight (g)] × 100
- VSI (%) = [gut weight (g) / fish weight (g)] × 100
- CF (%) = [perivisceral fat weight (g) / fish weight (g)] × 100.

### Physical characteristics, proximate composition and fatty acid profile of fillets

Nine fish per treatment were filleted, and the right fillets were weighed, packaged in a plastic bag and then refrigerated at + 4 °C. After 24 h at + 4 °C, the right fillets were gently dried with paper to remove excess moisture, and then weighed. Subsequently, the muscle pH (pH_24 h_) and flesh color were assessed on the inside portion of the cranial, medial and caudal region of each fillet. The pH_24_ measurement was performed using a Crison MicropH 2001 (Crison Instruments, Barcelona, Spain) equipped with a combined electrode and an automatic temperature compensator. The flesh color was analysed using a bench colorimeter Chroma Meter CR-400 (Konica Minolta Sensing Inc., Osaka, Japan). The results were expressed in terms of lightness (L*), redness (a*) and yellowness (b*) in the CIELAB color space model [28].

The water holding capacity was calculated as follows:

- Drip loss (DL; %) = [(raw fillet weight (g) − raw fillet weight after 24h (g)) / raw fillet weight (g)] × 100

The fillets were then individually vacuum-packaged in a plastic bag and stored at − 20 °C. After total freezing, the fillets were thawed at + 4 °C, removed from the bags, dried with paper, and weighed to calculate the thawed loss (TL) as follows:

- Thawing loss (TL; %) = [(raw fillet weight (g) − thawed fillet weight (g)) / raw fillet weight (g)] × 100

The same fillets were then vacuum-packaged in a plastic bag and cooked in a fish kettle for 10 min at 80 °C (core temperature of the fillets: 75 °C). After cooking, the bags were removed from the fish kettle and cooled in fresh water for 15 min to stop the cooking process. Then, the fillets were removed from the bags, dried with paper and weighed again to calculate the cooking loss (CL), as follows:

- Cooking loss (CL; %) = [(raw fillet weight (g) − cooked fillet weight (g)) / raw fillet weight (g)] × 100

Following cooking loss determination, a cooked fish sample (1.5 cm × 1.5 cm) from each fillet was sheared perpendicular to the fibre direction using the Instron 5543 Universal Testing Machine (Instron Corporation, Canton, Massachusetts, USA) equipped with a straight edged shear blade (crosshead speed of 30 mm/min). The maximum peak force recorded during the analysis was reported as Newton (N) shear force.

The nine left fillets per treatment were frozen, finely ground with a knife mill (Grindomix GM200; Retsch GmbH, Haan, Germany) and freeze-dried (Edwards MF 1000, Milan, Italy) to determine their proximate composition (DM, CP, EE, and ash), according to the same procedures implemented for feed analyses [23, 24]. The freeze-dried and ground samples of the fish fillet were also used to assess their FA composition. After dichloromethane-methanol extraction of total lipids from fillets, a basic saponification and a BF_3_ esterification were used for the determination of the fatty acid composition, adding tridecanoic acid as internal standard, as reported by Renna et al. [29]. FAME were separated using the same analytical instruments and temperature program previously reported for the FA analysis of feeds. Peaks were identified by injecting pure FAME standards as reported in Renna et al. [30]. The results were expressed as mg/100 g wet weight (ww). All chemical analyses were performed in duplicate.

### Morphometric investigation

Eight fish per treatment were submitted to morphometric and histopathological evaluation. Samples of anterior and posterior gut were excised and flushed with 0.9% saline solution to remove all the content. Liver samples were also collected. The collected samples were fixed in 10% buffered formalin solution, routinely embedded in paraffin wax blocks, sectioned at 5 μm thickness, mounted on glass slides and stained with Haematoxylin & Eosin. One slide per each intestinal segment was examined by light microscopy and captured with a Nikon DS-Fi1 digital camera (Nikon Corporation, Minato, Tokyo, Japan) coupled to a Zeiss Axiophot microscope (Carl Zeiss, Oberkochen, Germania) using 2.5× objective lens. NIS-Elements F software was used for image capturing.

Morphometric analysis was performed by Image®-Pro Plus software (6.0 version, Media Cybernetics, Maryland, USA) on 10 well-oriented and intact villi chosen from each gut segment. The evaluated morphometric index was villus height (Vh, from the villus tip to the crypt bottom). The observed histopathological findings were evaluated in all the organs using a semi-quantitative scoring system as follows: absent (score = 0), mild (score = 1), moderate (score = 2) and severe (score = 3). Gut histopathological findings were separately assessed for mucosa (inflammatory infiltrates) and submucosa (inflammatory infiltrates and Gut-Associated Lymphoid Tissue activation) for each segment. The total score of each gut segment was obtained by adding up the mucosa and submucosa scores. All the slides were blind assessed by two independent observers and the discordant cases were reviewed, using a multi-head microscope, until unanimous consensus was reached.

### Digestibility trial

In parallel with the growth trial, an *in vivo* digestibility experiment was performed to determine the apparent digestibility coefficients (ADC) of the diets. Two hundred and forty rainbow trout (mean individual body weight: 100.6 ± 8.53 g) were divided into twelve 250-L cylindroconical tanks (two replicates per treatment, twenty fish per tank) connected to the same open water system of the growth trial. After 14 days of acclimatization with the experimental diets, the fish were fed by hand to visual satiation two times per day (at 8:00 and 15:00 h), six days per week. The feces were collected daily from each tank for three consecutive weeks, using a continuous automatic device (Choubert’ system) as described by Palmegiano et al. [31]. To ensure the correct level of replications per treatment (i.e. *n* = 4), the experiment was conducted over two blocked events using the same batch of fish for both blocks. Before new fecal collection commenced, the fish were allowed to acclimatize to their new diet for a period of 10 days [32]. The feces were pooled within tank, kept frozen (− 20 °C) before being freeze dried and then refrigerated (+ 4 °C) until analysed. The ADCs were measured using the indirect acid-insoluble ash method. For this purpose, the fish were fed the same experimental diets of the growth trial, added with 1% Celite® (Fluka, St. Gallen, Switzerland) as an inert marker in substitution of 1% of wheat gluten meal. The ADC of DM, CP, EE, and GE were calculated as reported by Caimi et al. [19] and expressed as a percentage.

### Statistical analyses

Data were analysed using IBM SPSS Statistics v. 25.0 for Windows. One way-ANOVA or Kruskall Wallis tests were used to compare data among the experimental groups. The assumption of normality was checked using the Kolmogorov–Smirnov test. The assumption of homoscedasticity was assessed by Levene’s homogeneity of variance test. If such an assumption did not hold, the Brown-Forsythe statistic was applied to test the equality of group means instead of the F one. Pairwise multiple comparisons were performed to test the difference between each pair of means (Tukey’s test and Tamhane’s T2 in the cases of equal variances assumed or not assumed, respectively [one-way ANOVA], or Dunn’s test [Kruskall-Wallis test]). The results were expressed as the mean and pooled standard error of the mean (SEM) or median and interquartile range (IR), depending on data distribution. Significance was set at *P* < 0.05.

## Results

### Diets

As expected, the DM, ash, CP, EE and GE contents were comparable among the experimental diets (Table 1). Table 2 shows the AA composition of the BSF meal and the experimental diets. Leucine, valine and tyrosine were the most represented essential AA (EAA). Excepted for histidine, methionine and tyrosine, all EAA decreased with the increase of BSF inclusion. However, all diets covered the fish AA requirements. As far as the FA composition is concerned (Table 3), the concentration of total SFA increased at the increase of the insect meal in the diet. In particular, C12:0 showed a 42-fold higher concentration in BSF15 than in BSF0. Noticeable decreases were observed for total PUFA. Omega-3 FA drastically decreased and reached the absolute lowest concentration in the BSF15 diet.

### Growth performance

The mortality ranged from 0 (BSF0 and BSF12) to 2.1% (BSF15) and was not affected by the dietary treatment (Table 4). No differences were observed for the considered growth performance traits (*P* > 0.05).

**Table 4:** Effect of the dietary inclusion of BSF meal on growth performance of rainbow trout (*n* = 4)

	BSF0	BSF3	BSF6	BSF9	BSF12	BSF15	SEM	***P***-value
Mortality, %	0.00	1.04	1.04	1.04	0.00	2.08	0.512	0.708
iIBW, g	100.1	100.1	100.4	100.0	100.4	100.0	0.388	0.999
iFBW, g	278.8	282.3	284.9	278.4	272.9	267.0	2.06	0.179
iWG, g	178.8	182.3	184.6	178.5	172.6	167.3	3.536	0.818
SGR, %/d	0.84	0.84	0.84	0.83	0.82	0.78	0.010	0.675
FCR	1.08	1.09	1.09	1.12	1.13	1.18	0.013	0.398
PER	2.02	1.99	2.00	1.94	1.94	1.83	0.024	0.339

*Abbreviations*: *BSF Hermetia illucens*, *SEM* standard error of the mean, *iIBW* individual initial body weight, *iFBW* individual final body weight, *iWG* individual weight gain, *SGR* specific growth rate, *FCR* feed conversion ratio, *PER* protein efficiency ratio

### Somatic indexes, carcass yield and coefficient of fatness

The results concerning the somatic indexes, carcass yield and coefficient of fatness are reported in Table 5. The HSI showed an increasing trend with the increase of BSF meal inclusion in the diet. Specifically, HSI values were higher in BSF15 fish than BSF0 and BSF3 fish, while the other treatments showed intermediate values (*P* < 0.001). A similar trend was also observed for VSI, except for BSF0 that showed comparable values as BSF6, BSF9 and BSF12 (*P* < 0.01).

**Table 5:** Effect of the dietary inclusion of BSF meal on somatic indexes, carcass yield and coefficient of fatness of rainbow trout (*n* = 28)

	BSF0	BSF3	BSF6	BSF9	BSF12	BSF15	SEM	***P***-value
HSI	1.20^b^	1.22^b^	1.37^ab^	1.37^ab^	1.39^ab^	1.47^a^	0.019	0.000
VSI	11.2^ab^	11.0^b^	11.6^ab^	12.1^ab^	12.3^ab^	12.6^a^	0.143	0.008
CY	87.9	87.7	87.7	87.0	86.8	89.1	0.341	0.448
CF	3.65	3.64	3.64	3.82	3.84	3.24	0.093	0.622

*Abbreviations*: *BSF Hermetia illucens*, *SEM* standard error of the mean, *HSI* hepatosomatic index, *VSI* viscerosomatic index, *CY* carcass yield, *CF* coefficient of fatness. Different superscripts within a row indicate significant differences (*P* < 0.05)

### Physical characteristics, proximate composition and fatty acid profile of fillets

The dietary treatment did not affect either color or pH_24_ of the fillets (Table 6). The fillet’s physical characteristics were also unaffected by diet (Table 7).

**Table 6:** Effect of the dietary inclusion of BSF meal on flesh color and pH_24_ of cranial, medial and caudal regions of rainbow trout fillets (*n* = 9)

	BSF0	BSF3	BSF6	BSF9	BSF12	BSF15	SEM	***P***-value
Cranial region								
L*	42.3	46.1	43.5	45.0	43.2	43.4	0.799	0.788
a*	0.35	0.42	−0.32	2.06	−0.35	0.33	0.255	0.069
b*	6.25	8.06	6.32	8.17	6.17	6.60	0.258	0.051
pH_24_	6.49	6.31	6.38	6.32	6.37	6.42	0.020	0.101
Medial region								
L*	42.0	45.1	44.3	47.2	43.6	45.0	0.778	0.562
a*	1.57	3.73	1.58	2.70	2.88	1.87	0.316	0.292
b*	7.93	9.66	8.26	9.86	7.60	8.51	0.314	0.204
pH_24_	6.40	6.26	6.33	6.28	6.31	6.35	0.019	0.119
Caudal region								
L*	44.3	49.6	47.1	47.0	45.8	47.3	0.743	0.490
a*	2.95	2.14	2.93	3.92	4.81	2.31	0.579	0.784
b*	9.08	10.30	9.68	10.54	7.98	8.60	0.337	0.203
pH_24_	6.37	6.26	6.29	6.25	6.28	6.34	0.017	0.143

*Abbreviations*: *BSF Hermetia illucens*, L* lightness, a* redness, b* yellowness, *SEM *standard error of the mean

**Table 7:** Effect of the dietary inclusion of BSF meal on the physical characteristics of rainbow trout fillets (*n* = 9)

	BSF0	BSF3	BSF6	BSF9	BSF12	BSF15	SEM	***P***-value
Drip loss, %	2.89	3.32	2.76	3.22	2.80	3.04	0.114	0.658
Thawed loss, %	9.50	9.47	8.98	10.52	10.18	9.35	0.240	0.470
Cooking loss, %	16.6	17.6	16.6	18.8	18.3	17.7	0.373	0.439
Shear force, N	28.8	26.0	21.1	25.8	26.7	22.71	1.142	0.434

*Abbreviations*: *BSF Hermetia illucens*, *SEM* standard error of the mean

The DM, CP and EE contents of the fillets did not show differences among the dietary treatments. The ash content was higher in BSF0 compared to BSF9, BSF12 and BSF15, while the fish fed with BSF3 and BSF6 showed intermediate values.

As for FA contents, total SFA and total MUFA showed a clear increasing trend, while a decreasing trend was observed for total PUFA, as the level of BSF larva meal increased in the diet (Table 8). Consequently, the Σ PUFA / Σ SFA ratio of the fillets progressively decreased, ranking in the following order: BSF0 = BSF3 = BSF6 > BSF9 > BSF12 > BSF15. The fish fed with BSF15 showed a higher content of total SFA when compared to the fish fed with BSF0 (+ 29%), while the fish fed with the other treatments showed intermediate values (*P* < 0.05). Regarding individual SFA, C12:0 was about 19-fold higher in BSF15 when compared to BSF0 (1.73 vs. 0.09 g/100 g TFA, respectively; *P* < 0.001). An increasing trend was also observed for C14:0 with the dietary increase of the insect meal, while other individual SFA (i.e., C16:0, C17:0, C18:0 and C20:0) only showed higher values in BSF15 when compared to the other treatments. Various branched chain fatty acids (BCFA) were detected. The rates of all of them, with the exception of C18 *iso*, were significantly affected by the diet. The majority of BCFA showed the absolute highest content when the fish were fed the BSF15 diet. Total MUFA and C18:1 *c-*9 were about 1.3-fold higher in BSF15 when compared to BSF0. As previously described for individual SFA and BCFA, some individual MUFA (i.e., C14:1 *c-*9 – which coeluted with C15:0 in the chromatograms –, total C18:1 *t* and C18:1 *c-*11) showed higher values in BSF15 when compared to the other treatments. The observed decrease of total PUFA was substantial (− 58% in BSF15 when compared to BSF0) and regarded both total n-3 and total n-6 FA (*P* < 0.001). The decrease was more marked for FA belonging to the n-3 (− 81% considering total n-3 FA) than the n-6 (− 43% considering total n-6 FA) series. Consequently, the Σ n-3 / Σ n-6 FA ratio also significantly decreased while increasing BSF meal in the diet, following the order BSF0 > BSF3 = BSF6 = BSF9 > BSF12 > BSF15. All individual n6 FA showed significantly lower values in BSF15 when compared to the other treatments. Similar results were obtained for C18:3 n-3 and C20:3 n-3, while the decreasing trend was gradual for long-chain n-3 PUFA (C20:5 n-3, C22:5 n-3 and C22:6 n-3).

**Table 8:** Effect of the dietary inclusion of BSF meal on fillet proximate composition (g/100 g ww) and fatty acid profile (g/100 g of TFA) of rainbow trout (*n* = 9)

	BSF0	BSF3	BSF6	BSF9	BSF12	BSF15	SEM	***P***-value
Proximate composition								
DM	25.8	26.8	27.2	25.5	26.1	26.6	0.303	0.643
CP	19.2	20.0	18.3	19.2	19.6	20.5	0.323	0.493
EE	4.44	4.97	5.34	4.74	5.13	4.76	0.223	0.897
Ash	2.06^a^	1.68^ab^	1.54^ab^	1.43^b^	1.28^b^	1.22^b^	0.065	0.010
Fatty acid composition								
C12:0	0.09^e^	0.28^de^	0.44^d^	0.75^c^	1.16^b^	1.73^a^	0.081	0.000
C13 *iso*	0.07^ab^	0.07^ab^	0.06^ab^	0.07^a^	0.05^ab^	0.04^b^	0.003	0.024
C14:0	2.93^c^	3.00^bc^	2.91^c^	3.16^bc^	3.35^b^	4.36^a^	0.078	0.000
C15 *iso*	0.07^b^	0.07^b^	0.07^b^	0.06^b^	0.07^b^	0.09^a^	0.001	0.000
C15 *aiso*	0.02^b^	0.02^b^	0.02^b^	0.02^b^	0.03^ab^	0.04^a^	0.002	0.000
C14:1 *c-*9 + C15:0	0.33^b^	0.33^b^	0.32^b^	0.34^b^	0.34^b^	0.43^a^	0.007	0.000
C16:0	18.6^b^	19.1^b^	18.8^b^	19.6^b^	20.2^b^	25.4^a^	0.361	0.000
C17 *iso*	0.21^b^	0.22^b^	0.21^b^	0.20^b^	0.22^b^	0.28^a^	0.005	0.000
C17 *aiso*	0.43^cd^	0.42^d^	0.42^d^	0.50^bc^	0.52^b^	0.64^a^	0.013	0.000
C16:1 *c-*9	4.88^c^	5.14^bc^	5.03^bc^	5.29^bc^	5.52^b^	6.31^a^	0.085	0.000
C17:0	0.29^b^	0.28^b^	0.29^b^	0.27^b^	0.28^b^	0.40^a^	0.008	0.000
C18 *iso*	0.12	0.11	0.10	0.12	0.12	0.12	0.004	0.421
C17:1 *c-*9	0.19	0.18	0.20	0.23	0.21	0.21	0.006	0.217
C18:0	5.11^b^	5.13^b^	5.10^b^	5.32^b^	5.44^b^	6.53^a^	0.078	0.000
C18:1 *t-*6–10	0.09^b^	0.10^b^	0.09^b^	0.10^b^	0.11^ab^	0.12^a^	0.002	0.000
C18:1 *t-*11–12	0.03	0.03	0.02	0.02	0.02	0.03	0.001	0.104
C18:1 *t-*13–14 + *c-*6–8	0.12^b^	0.12^b^	0.11^b^	0.13^b^	0.12^b^	0.16^a^	0.003	0.000
C18:1 *c-*9	23.3^d^	24.0^cd^	24.9^bcd^	25.4^bc^	26.6^b^	31.3^a^	0.397	0.000
C18:1 *c-*11	2.88^b^	2.93^b^	2.92^b^	2.93^b^	2.99^b^	3.50^a^	0.038	0.000
C18:2 n-6	21.0^a^	21.6^a^	21.8^a^	20.9^a^	20.7^a^	12.3^b^	0.513	0.000
C18:3 n-3	2.51^a^	2.62^a^	2.51^a^	2.41^a^	2.22^a^	0.93^b^	0.089	0.000
C18:3 n-6	0.32^a^	0.31^a^	0.34^a^	0.36^a^	0.29^a^	0.12^b^	0.014	0.000
C20:0	0.16^b^	0.17^b^	0.18^b^	0.18^b^	0.18^b^	0.23^a^	0.004	0.000
C20:1 *c-*9	1.17^b^	1.15^b^	1.20^b^	1.22^b^	1.26^ab^	1.44^a^	0.024	0.003
C20:1 *c-*11	0.17^a^	0.16^ab^	0.15^ab^	0.15^ab^	0.14^b^	0.05^c^	0.006	0.000
C20:2 n-6	0.98^a^	0.93^a^	1.01^a^	0.96^a^	0.95^a^	0.66^b^	0.025	0.000
C20:3 n-3	0.15^a^	0.15^a^	0.16^a^	0.14^a^	0.14^a^	0.07^b^	0.005	0.000
C20:3 n-6	0.63^a^	0.59^a^	0.64^a^	0.70^a^	0.60^a^	0.20^b^	0.025	0.000
C20:4 n-6	0.75^a^	0.72^a^	0.73^a^	0.76^a^	0.62^a^	0.22^b^	0.031	0.000
C20:5 n-3	2.96^ab^	3.14^a^	2.68^ab^	2.53^b^	1.82^c^	0.54^d^	0.128	0.000
C22:0	0.10	0.10	0.12	0.12	0.11	0.11	0.002	0.055
C22:1 n-9	0.14	0.14	0.15	0.16	0.17	0.17	0.006	0.517
C22:5 n-3	0.92^ab^	1.03^a^	0.92^ab^	0.80^b^	0.55^c^	0.12^d^	0.046	0.000
C22:6 n-3	8.19^a^	5.56^b^	5.35^bc^	4.18^cd^	2.97^d^	1.11^e^	0.326	0.000
Σ SFA	27.7^b^	28.4^ab^	28.2^ab^	29.7^ab^	31.0^ab^	35.7^a^	0.821	0.043
Σ BCFA	0.91^bc^	0.91^bc^	0.86^c^	0.97^b^	1.00^b^	1.22^a^	0.018	0.000
Σ MUFA	33.0^d^	34.0^cd^	34.8^bcd^	35.6^bc^	37.2^b^	43.3^a^	0.519	0.000
Σ PUFA	38.4^a^	36.7^ab^	36.1^ab^	33.8^bc^	30.8^c^	16.3^d^	1.089	0.000
Σ PUFA / Σ SFA	1.39^a^	1.29^a^	1.28^a^	1.14^b^	1.00^c^	0.39^d^	0.047	0.000
Σ n-3	14.7^a^	12.5^b^	11.6^bc^	10.1^c^	7.71^d^	2.78^e^	0.559	0.000
Σ n-6	23.7^a^	24.2^a^	24.5^a^	23.7^a^	23.1^a^	13.5^b^	0.590	0.000
Σ n-3 / Σ n-6	0.62^a^	0.51^b^	0.47^b^	0.43^b^	0.34^c^	0.19^d^	0.020	0.000

*Abbreviations*: *BSF Hermetia illucens*, *ww* wet weight, *TFA* total fatty acid, *SEM* standard error of the mean, *DM* dry matter, *CP* crude protein, *EE* ether extract, *c cis*, *t trans*, *SFA* saturated fatty acids, *BCFA* branched chain fatty acids, *MUFA* monounsaturated fatty acids, *PUFA* polyunsaturated fatty acids. Values are reported as mean of duplicate analyses. Different superscripts within a row indicate significant differences (*P* < 0.05)

### Morphometric investigation

No differences were found for morphometry at the anterior gut, whereas villi were higher in the posterior gut of BSF6 and BSF9 groups when compared to BSF3 (Table 9).

**Table 9:** Effect of the dietary inclusion of BSF meal on morphometric traits of anterior and posterior gut of rainbow trout (*n* = 8)

	BSF0	BSF3	BSF6	BSF9	BSF12	BSF15	SEM	***P***-value
Vh anterior gut	0.54	0.51	0.54	0.50	0.54	0.49	0.015	0.906
Vh posterior gut	0.66^abc^	0.53^c^	0.66^a^	0.63^ab^	0.63^abc^	0.53^bc^	0.013	0.007

*Abbreviations*: *BSF Hermetia illucens*, *SEM* standard error mean, *Vh* villus height. Different superscripts within a row indicate significant differences (*P* < 0.05)

Regarding the histopathological alterations of liver, absent to mild multifocal lymphoplasmacytic inflammatory infiltrates were observed with absent/mild vacuolar degeneration (Table 10). The anterior and posterior gut showed from absent to mild mucosal lymphoplasmacytic infiltration. However, BSF meal inclusion did not affect the severity of the observed histopathological alterations (*P* > 0.05).

**Table 10:** Effect of the dietary inclusion of BSF meal on histopathological alterations of liver and gut of rainbow trout (*n* = 8)

	BSF0	BSF3	BSF6	BSF9	BSF12	BSF15	***P***-value
Liver							
Inflammation, median (IR)	0.5 (0.0–1.0)	0.0 (0.0–0.4)	0.0 (0.0–0.0)	0.0 (0.0–1.0)	0.0 (0.0–0.0)	0.0 (0.0–0.0)	0.057
Degeneration, median (IR)	0.0 (0.0–0.4)	0.0 (0.0–0.5)	0.25 (0.0–1.0)	1.25 (0.5–1.9)	0.25 (0.0–1.0)	0.0 (0.0–1.0)	0.071
Gut							
Anterior, median (IR)	0.0 (0.0–0.0)	0.0 (0.0–0.5)	0.0 (0.0–0.9)	0.0 (0.0–0.0)	0.0 (0.0–0.8)	0.0 (0.0–0.0)	0.180
Posterior, median (IR)	0.0 (0.0–0.8)	0.0 (0.0–0.0)	0.0 (0.0–0.0)	0.0 (0.0–0.0)	0.0 (0.0–0.0)	0.0 (0.0–0.0)	0.345

*Abbreviations*: *BSF Hermetia illucens*, *IR* interquartile range

### Digestibility trial

The apparent digestibility of DM, CP, EE and GE was not influenced by diet (*P* > 0.05) (Table 11). The ADC of DM ranged between 86.8% (BSF6) and 87.7% (BSF9). Overall, the ADC of CP and EE were higher than 94% and 98%, respectively. Results concerning ADC of GE ranged between 92.1% (BSF9) and 93.1% (BSF15).

**Table 11:** Apparent digestibility coefficients (ADC) of the experimental diets *(n* = 4)

	BSF0	BSF3	BSF6	BSF9	BSF12	BSF15	SEM	***P***-value
ADC_DM_, %	87.3	87.1	86.8	87.7	86.9	87.5	0.332	0.988
ADC_CP_, %	94.7	94.2	94.4	94.6	94.2	94.6	0.163	0.967
ADC_EE_, %	98.8	98.7	98.7	98.8	98.6	98.6	0.054	0.903
ADC_GE_, %	92.9	92.5	92.7	92.1	92.6	93.4	0.240	0.947

*Abbreviations*: *BSF Hermetia illucens*, *SEM* standard error of the mean, *DM* dry matter, *CP* crude protein, *EE* ether extract, *GE* gross energy. Different superscript letters within a row indicate significant differences (*P* < 0.05)

## Discussion

### Growth performance

Starting from the first day of trial, all the fish promptly accepted all the experimental diets. After 131 days of trial, there were no significant differences among the groups in terms of growth performance and feed utilization. The results obtained in the present study showed that, in current typical commercial diets for trout, it is possible to substitute up to the 50% of FM with a partially defatted BSF larva meal (corresponding to a maximum of 15% dietary inclusion level) without adverse effects on the fish growth performance. Such results are consistent with previous trials in which other fish species were fed diets with inclusion levels up to 20% of full-fat [33], partially defatted [21, 34] and highly defatted BSF meals [19, 35]. Indeed, inclusion levels of BSF meal higher than 20% can induce a stress response in rainbow trout [36]. Published studies show that dietary inclusion levels of about 33% and 26% of full-fat BSF meals [16, 37] or of a partially defatted BSF meal [15] determined a worsening of the WG and feed utilization. Similar results were also observed in meagre (*Argyrosomus regius*) when fed increasing amounts of a partially defatted BSF meal [20]. However, other trials showed that it could be possible to include up to 40% of a partially defatted BSF larva meal [14] or up to 50% of a full-fat BSF prepupae meal [13] in substitution of FM, without negative effects on the growth performance of adult and juvenile rainbow trout, respectively.

### Somatic indexes, carcass yield and coefficient of fatness

In literature, HSI values between 1% and 2% (as obtained in the current trial) are indicators of proper liver function [38]. Published data on the effect of insect meals on HSI values in rainbow trout are inconsistent. Similarly to what obtained in our trial, Dumas et al. [15] and Chemello et al. [22] also reported an increase of HSI in rainbow trout fed with partially defatted BSF and *Tenebrio molitor* meals, respectively. On the contrary, Sealey et al. [37] recorded a decrease in HSI in fish fed with diets characterized by increasing levels of BSF meal. Renna et al. [14] observed that inclusion levels up to 40% of a partially defatted BSF larva meal did not influence the HSI in rainbow trout. The dissimilarity of results found in literature could be related to several factors, such as the insect species and the nutritional composition of the insect meal, whereas the chemical composition of BSF meal, especially its fat content and FA profile, could be influenced by the rearing substrate of the larva [9, 39, 40] and the defatting method [15, 41]. In addition, results can also differ due to fish species, size and physiological status.

### Physical characteristics, proximate composition and fatty acid profile of fillets

The pH of fish fillets provides a measure of the stress status of the fish and the flesh freshness. Changes in pH could be due by multiple factors, such as high density in tank, stress in pre-killing phase and dietary treatments [42]. An increase of the pH during storage could also reflect the production of bacterial metabolites and therefore a decrease of the shelf life of the product [43]. In the currently trial, the pH of the fillets was not affected by diet, and the recorded values are in line with those previously reported for rainbow trout fed with BSF larva meals [14, 44] and *Tenebrio molitor* larva meals [38]. The results obtained in our study also showed that an inclusion of partially defatted BSF meal up to 15% did not influence the color of the raw fillets. Such results confirm those recently obtained by Secci et al. [44] in rainbow trout fed up to 40% of partially defatted BSF larva meal. The lack of influence of BSF meal on fillet color should be considered as an advantage. In fact, color is an important quality parameter of the fillet, as it directly influences the perception of freshness, being also used by consumers to evaluate product quality [45].

We observed no differences in terms of fillets DL, TL, CL and SF among the experimental groups. Similarly, Secci et al. [44] and Borgogno et al. [46] reported no significant variations for the SF and CL when rainbow trout were fed with BSF meals. In our trial, both SF and CL showed higher values when compared to those obtained by the above-mentioned authors. Such difference may be due to different factors, such as sampling procedure, preservation and storage of samples and analytical methods used.

The lack of effects of BSF meal on fillet DM, CP and EE contents confirms previous findings obtained by Renna et al. [14], Mancini et al. [47] and Reyes et al. [48], when BSF larva meals were included in rainbow trout diets up to inclusion levels of 25%. In our trial, the ash content of the fillets showed a decreasing trend following increasing levels of BSF larva meal inclusion in the diet. When compared to FM, BSF meal showed noticeably lower phosphorous levels [49]. Such difference may have determined the observed significant decreases in fillet ash content at increasing BSF levels in the diet. However, in young grass carp (*Ctenopharyngodon idella* Valenciennes), Wen et al. [50] observed decreased levels of ash in fillets while increasing the dietary level of available phosphorous. As no information can be found in literature on rainbow trout, further studies are needed to clearly understand the obtained results.

Fish contain high amounts of long-chain n-3 PUFA, well known to exert beneficial effects on human health [51]. Usually, the FA composition of fish fillets reflects that of the administered diet [14]. The FA content and composition of insect larvae (and derived meals) depend on the considered insect species, rearing substrate, developmental stage and processing (e.g., defatting methods) [52]. Differently from FM, insects are generally rich in SFA and poor in PUFA [53]. In particular, BSF larval fat consists mainly of C12:0 and other SFA [9, 52], as confirmed by the analyses of the tested BSF meal. Even if the BSF meal used in this trial was partially defatted (EE: 7.0 g/100 g as fed), this led to a noticeable increase of C12:0 in the BSF-containing diets when compared to the FM-control diet (BSF0), as already observed by other authors [14, 20]. In recent trials where high inclusion levels of insect meals were evaluated in aquafeed for various fish species, significant alterations of the FA composition of fish whole body and fish fillets were observed [21, 47, 54]. The most frequently reported modifications were increasing levels of SFA associated with reductions of PUFA contents, particularly when considering long-chain PUFA of the n-3 series (i.e., C20:5n-3 and C22:6n-3), which also led to undesirable decreases of the PUFA/SFA and n-3/n-6 PUFA ratios of the product [19, 20, 44]. Our results confirm such findings and, in addition, clearly show that the FA composition of trout fillets could be negatively affected even in case of low inclusion levels of BSF meal as a replacement of FM in typical commercial diets.

### Morphometric investigation

Regardless the dietary treatment, Vh showed a proximo-distal increasing gradient from anterior to posterior gut. In literature, the majority of the studies conducted in mammals and poultry reported a proximo-distal decreasing gradient from anterior to posterior gut [55]. This is due to the different intensity of nutrient digestion and absorption processes along the gut. Only few publications are available on the morphometry of rainbow trout and they mainly concentrated on the anterior gut [56] as it is the most important site for nutrient absorption, receiving physical, chemical and hormonal stimuli caused by the presence of the diet in the lumen [57, 58]. The lack of differences for Vh in the anterior gut among the dietary treatments is a positive finding and it is in accordance with Renna et al. [14] who did not record any morphological changes in the intestine of trout fed up to 40% of BSF meal. Moreover, the unaffected morphometry of the anterior gut could also explain the unchanged growth performances and diet digestibility recorded for the trout of the present study, suggesting good nutrient absorption and utilization with BSF meal dietary inclusion levels up to 15%. On the contrary, BSF meal influenced Vh in the posterior gut, being lower in BSF3 diet compared to the other treatments. This is one of the first studies describing posterior gut in trout and further study are needed to better investigate this upward trend. Regarding the histopathological alterations of liver and gut, they varied from absent to mild in all the organs. The absence of adverse effects related to dietary BSF meal inclusion observed in liver and gut of the present study is in agreement with published literature [36, 59, 60].

### Digestibility trial

The ADC values of nutrients and energy were high and comparable among the experimental groups. Such results suggest that BSF larva meal is well digested by rainbow trout and its inclusion up to 15% in the diet does not adversely affect the fish growth performance. In fact, studies conducted on rainbow trout with BSF larva meal [14] and *Tenebrio molitor* larva meal [22, 61], showed that dietary inclusion levels up to 25% did not affect the apparent digestibility of nutrients and energy.

## Conclusions

The findings of this study suggest that partially defatted black soldier fly larva meal can be considered a suitable ingredient in low fishmeal-based diets for rainbow trout. In this study, we did not observe any adverse effect on growth performance, somatic indexes, fillet physical quality parameters and chemical composition, and diets digestibility. However, the FA composition of fillets worsened while increasing the level of BSF meal in the diets. In particular, a decrease of PUFA, and an increase of total SFA and total MUFA was observed. The commercial use of insect meal for aquaculture feed is a good approach to reduce the environmental impact and support a sustainable increase in aquaculture production.

## Data Availability

The datasets analysed in the current study are available from the corresponding author on request.

## Abbreviations

ADC: Apparent digestibility coefficient
ADF: Acid detergent fiber
BCFA: Branched chain fatty acids
BSF: *Hermetia illucens*
*c*: *cis*
CF: Coefficient of fatness
CL: Cooking loss
CP: Crude protein
CY: Carcass yield
DL: Drip loss
DM: Dry matter
EE: Ether extract
FA: Fatty acid
FAME: Fatty acid methyl ester
FBW: Final body weight
FCR: Feed conversion ratio
FM: Fish meal
GE: Gross energy
HSI: Hepatosomatic index
IBW: Initial body weight
IR: Interquartile range
MUFA: Monounsaturated fatty acids
nd: Not detected
PAPs: Processed animal proteins
PER: Protein efficiency ratio
PUFA: Polyunsaturated fatty acids
SEM: Standard error of the mean
SF: Shear force
SFA: Saturated fatty acids
SGR: Specific growth rate
*t*: *trans*
TFA: Total fatty acids
TL: Thawed loss
Vh: Villus height
VSI: Viscerosomatic index
WG: Weight gain
ww: Wet weight
